# *De novo* assembly and characterization of the *Hucho taimen* transcriptome

**DOI:** 10.1002/ece3.3735

**Published:** 2017-12-21

**Authors:** Guang‐Xiang Tong, Wei Xu, Yong‐Quan Zhang, Qing‐Yu Zhang, Jia‐Sheng Yin, You‐Yi Kuang

**Affiliations:** ^1^ Heilongjiang River Fisheries Research Institute of Chinese Academy of Fishery Sciences Daoli District, Harbin Heilongjiang Province China

**Keywords:** comparative transcript analysis, *Hucho taimen*, microsatellite markers, positive selection, RNA‐Seq

## Abstract

Taimen (*Hucho taimen*) is an important ecological and economic species that is classified as vulnerable by the IUCN Red List of Threatened Species; however, limited genomic information is available on this species. RNA‐Seq is a useful tool for obtaining genetic information and developing genetic markers for nonmodel species in addition to its application in gene expression profiling. In this study, we performed a comprehensive RNA‐Seq analysis of taimen. We obtained 157 M clean reads (14.7 Gb) and used them to *de novo* assemble a high‐quality transcriptome with a N50 size of 1,060 bp. In the assembly, 82% of the transcripts were annotated using several databases, and 14,666 of the transcripts contained a full open reading frame. The assembly covered 75% of the transcripts of Atlantic salmon and 57.3% of the protein‐coding genes of rainbow trout. To learn about the genome evolution, we performed a systematic comparative analysis across 11 teleosts including eight salmonids and found 313 unique gene families in taimen. Using Atlantic salmon and rainbow trout transcriptomes as the background, we identified 250 positive selection transcripts. The pathway enrichment analysis revealed a unique characteristic of taimen: It possesses more immune‐related genes than Atlantic salmon and rainbow trout; moreover, some genes have undergone strong positive selection. We also developed a pipeline for identifying microsatellite marker genotypes in samples and successfully identified 24 polymorphic microsatellite markers for taimen. These data and tools are useful for studying conservation genetics, phylogenetics, evolution among salmonids, and selective breeding for threatened taimen.

## 1 INTRODUCTION

The genus *Hucho* belongs to the Salmonidae family and includes four species that are endangered. Taimen (*Hucho taimen*) is a strictly freshwater member of *Hucho* that is distributed widely from the Danube drainage basin to the Pacific Ocean in terms of longitude and from the Arctic Ocean to Mongolia and northern China in terms of latitude. In recent decades, overexploitation, hydropower dams, and pollution have dramatically diminished the habitat of this species (Gilroy et al., 2010; Zolotukhin, 2013), and its population has decreased approximately 37.3% worldwide (Hogan & Jensen, 2013). Taimen is also a rare endemic species in China. In the 1950s, this fish had a wide distribution from Northwest China to Northeast China, and the harvest was abundant. At present, however, only a small population remains in Kanas Lake in Xinjiang Province and the Amur River in Heilongjiang Province, and the estimated abundance in China has decreased by 80% over the past three generations (Hogan & Jensen, 2013). Therefore, taimen has been listed as an endangered species in China since 1998 (Yue & Chen, 1998) and has been included on the China Species Red List since 2004 (Wang & Xie, 2004). The global endangered status of taimen was re‐evaluated in 2013, and it was then classified as vulnerable on the IUCN Red List of Threatened Species (Hogan & Jensen, 2013).

Taimen also has great potential to become an excellent aquaculture species because it is the largest salmonid and can grow to 55–66 kg in body weight and 160–170 cm in total length (Andreji & Stráňai, 2013). Moreover, taimen is one of the fastest growth species among salmonids (Andreji & Stráňai, 2013), and its body weight increases at an approximately linear rate below 10 years of age (Andreji & Stráňai, 2013). For conservation and exploitation of this species, artificial propagation and fry rearing have been successfully developed, and the species has been cultured widely in China (Li, Wang, Liu, Yin, & Lu, 2016; Wang et al., 2016). During aquaculture activity, taimen presents higher bacterial disease resistance than rainbow trout under the same conditions (Li et al., 2016; Wang et al., 2016).

Although taimen is considered an ecologically and economically valuable species, few genetic resources are available for studies of its conservation genetics, phylogenetics, and selective breeding. Only approximately 450 taimen nucleotide recodes are available in GenBank (searched in 9 May 2017) and most are mitochondrial genes. Tong, Kuang, Yin, Liang, and Sun (2006) and Wang, Kuang, Tong, and Yin (2011) developed microsatellite markers; and Wang, Zhang, Yang, and Song (2011) and Balakirev, Romanov, Mikheev, and Ayala (2016) sequenced the complete mitochondrial genome. RNA‐Seq is an excellent technology for studying phylogenetics and evolution and developing SSR and SNP markers for nonmodel species (Cahais et al., 2012; Ekblom & Galindo, 2010; Qian, Ba, Zhuang, & Zhong, 2014). In this study, we used RNA‐Seq to construct the transcriptome of taimen and develop SSR and SNP markers, and we also performed a systematic cross‐species comparative analysis for taimen and other salmonids.

## 2 MATERIALS AND METHODS

### 2.1 Sample collection and RNA isolation

**Figure 1:**
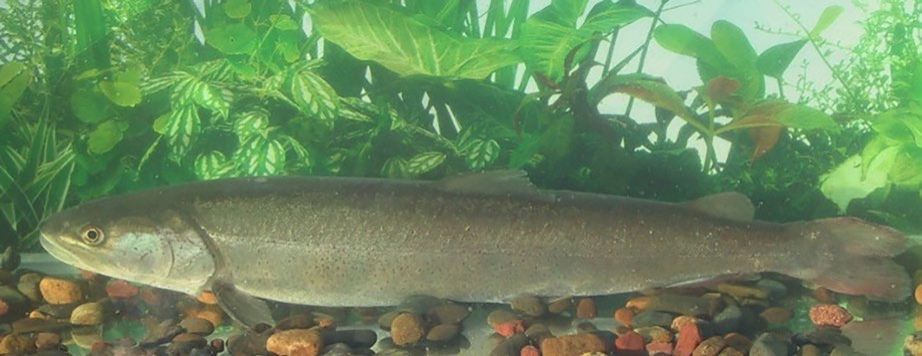
Yong fish (age 3+) of *Hucho taimen*. Photographed by Wei Xu, with permission

The RNA‐Seq analysis was performed on first‐generation offspring (Figure 1) artificially reproduced from wild stock, and these fish were collected from the Bohai Station of the Heilongjiang River Fisheries Research Institute (HRFRI) of the Chinese Academy of Fishery Sciences. To obtain the whole reference transcriptome, 10 individuals were collected, including two individuals at age 4+, six individuals at age 2+, and two individuals at age 1+. Twelve organs from each individual, including the skin, muscle, eyes, brain, spleen, kidney, intestines, stomach, liver, testes, ovaries, and gills, were dissected for RNA extraction, and all tissue samples were stored in RNALater solution (Qiagen, CA, USA) for transport. Total RNA was extracted using an RNeasy kit (Qiagen, CA, USA) and treated with DNaseI (Invitrogen, CA, USA) to remove genomic DNA. After a quality examination using a Bioanalyzer 2100 system (Agilent, CA, USA) and quantitation using a NanoDrop 8000 system (Thermo Fisher Scientific Inc., CA, USA), equal quantities of total RNA with RIN (RNA integrity number) ≥7.0 from each organ of each individual were mixed to construct the RNA‐Seq library. All experiments involving the handling and treatment of fish in this study were approved by the Animal Care and Use committee of the HRFRI of the Chinese Academy of Fishery Sciences.

### 2.2 cDNA library construction and sequencing

Two normalized cDNA libraries were constructed using an RNA‐Seq assay for transcriptome sequencing. One paired‐end cDNA library was generated from the pooled total RNA of 12 tissues from one individual at age 4+, and another library was generated from the pooled total RNA of 12 tissues from the other nine individuals. The paired‐end cDNA libraries were prepared using a TruSeq RNA prep Kit (Illumina, CA, USA) according to the Illumina protocols. The insert size was approximately 200 bp, and the two libraries were normalized with a DSN normalization kit (Illumina, CA, USA). The normalization libraries were sequenced on an Illumina HiSeq2000 system in the 100‐bp pair‐end mode. The cDNA library construction and sequencing were performed by a commercial service company (Genergy Inc, Shanghai, China).

### 2.3 De novo assembly

The transcriptome sequences were assembled using the Trinity package (Grabherr et al., 2011; Haas et al., 2013). Before assembly, low‐quality reads were filtered from the raw reads using Trimmomatic (Bolger, Lohse, & Usadel, 2014) with the parameters LEADING:30 TRAILING:30 SLIDINGWINDOW:4:20 MINLEN:50. The clean reads from the two pooled libraries were merged and in silico normalized using the Trinity package with default parameters to reduce the running time and memory consumption. A parameter kmer size of 25 and a depth of at least two kmer were used for assembly with the Trinity package. The contigs resulting from Trinity were further fed to the TGI clustering Tool (version 2.1) (Pertea et al., 2003) to process alternative splicing and redundant sequences.

### 2.4 Assembly assessment

A gold standard is not available to assess the quality of a de novo assembly transcriptome for a nonmodel species. We adopted general methods suggested by Trinity (Haas et al., 2013) and Fu et al. (Fu & He, 2012) to assess the assembly quality. The assessment method consisted of five criteria: read composition of the assembly, number of full‐length protein‐coding transcripts, quality of the assembled transcript sequences, completeness, and gene coverage. First, Bowtie2 (Langmead & Salzberg, 2012) was used to map the clean reads to the assembly, and a Perl script in the Trinity package was then used to summarize the properly mapped reads. Generally, a vast number of reads were mapped back to the assembly, and among them, approximately 70%–80% of the mapped reads were proper pairs. Second, the full‐length protein‐coding transcripts were characterized using the Blastx or Blastp tool, and the Swiss‐Prot protein database and several species’ (including zebrafish, threespine stickleback, Atlantic salmon, rainbow trout, medaka, fugu, and green spotted puffer) whole‐genome‐predicted protein sequences (Table S1) were used as references to search the conserved regions. A transcript was classified as a full‐length protein‐coding transcript if it contained a complete coding region (Fu & He, 2012) according to the Blast alignments. Third, the assembled sequence quality was assessed. Thirteen mitochondrial protein‐coding genes from the mitochondrial genome of taimen and 20 mRNA sequences from GenBank were used to assess the assembled sequence quality, and the sequences were aligned to the assembly transcriptome with Blastn. Then, the identity percent and coverage were calculated. Furthermore, transcriptome completeness was assessed based on the conserved ortholog content using BUSCO (Simão, Waterhouse, Ioannidis, Kriventseva, & Zdobnov, 2015), which uses evolutionarily informed expectations of gene content from near‐universal single‐copy orthologs to quantitatively measure transcriptome completeness. Single‐copy orthologs from the Actinopterygii lineage were adopted for the assessment. Finally, gene coverage was assessed by aligning the assembled taimen transcriptome to the genomes of Atlantic salmon (Lien et al., 2016) and rainbow trout (Berthelot et al., 2014) using GMAP (Wu & Watanabe, 2005). We used the options ‐x and ‐n 0 to report chimeric transcripts and best alignments. After removing the chimeric transcripts and filtering out the low query coverage (<70%), the GMAP alignments were grouped according to the overlapping coordinates on the Atlantic salmon or rainbow trout genome. If a gene in the Atlantic salmon or rainbow trout genome was covered by the grouped alignment, then the gene was counted.

### 2.5 Functional annotation

We used TransDecoder (https://transdecoder.github.io/) to predict the open reading frames (ORFs) and translating proteins, and homology searches against pFam and Uniprot databases were performed as supporting evidence for the ORFs. The ORFs with <30 amino acids were discarded. We also used GeneMarkS‐T (Tang, Lomsadze, & Borodovsky, 2015) to predict the ORFs to complement the TransDecoder predicted ORFs, and used Cd‐hit package (Li & Godzik, 2006) to merge the two datasets. BLAST tools were used to search transcripts and predicted proteins for homologs against protein databases, including the NR, Swiss‐Prot, Refseq, and Ensembl‐annotated protein databases of several species (Zebrafish, Stickleback, Medaka, Fugu, and Tetraodon) as well as the whole‐genome‐predicted protein databases of rainbow trout (Berthelot et al., 2014) and Atlantic salmon (Lien et al., 2016). Blastn was also used to search for nucleotide databases (NT, Refseq, and whole‐genome‐annotated mRNA sequences of the species listed above). During the BLAST search, only the best significant hits were retained for each transcript. The conserved protein domain was analyzed using the Pfam database. Signal peptides and transmembrane domains were analyzed using SignalP and tmHMM software, respectively. For homologous searching, a cutoff *e*‐value of 1e‐10 (for nucleotide databases) or 1e‐5 (for protein databases) was used. Furthermore, these analysis results were integrated with Trinotate (https://trinotate.github.io/) to perform biological function annotations and Gene Ontology (GO) classifications. We also used Interproscan to search the protein conserved domains and perform GO classifications (Jones et al., 2014). KEGG annotation was performed using GhostKOALA (Kanehisa, Sato, & Morishima, 2016).

### 2.6 Comparative analysis among Salmonidae

We first used the Conditional Reciprocal Best Blast (CRB‐BLAST) (Aubry, Kelly, Kümpers, Smith‐Unna, & Hibberd, 2014) algorithm to identify orthologs between taimen and Atlantic salmon (Lien et al., 2016) and rainbow trout (Berthelot et al., 2014). Compared with the traditional Reciprocal Best Hit (RBH) algorithm, the CRB‐BLAST algorithm learned an appropriate *e*‐value cutoff by fitting a function to the distribution of alignment *e*‐values over the sequence length to assign orthologs, and this process generated more accurate orthologs for de novo transcriptome assembly (Aubry et al., 2014). The ortholog pairs were aligned using MUSCLE, and the pairwise Ka/Ks values between taimen and Atlantic salmon and rainbow trout were calculated using the CodeML (pairwise mode) of PAML (Yang, 2007).

We then searched for duplicate genes using the reciprocal self‐BLAST hit method. After filtering out gene variations (alternative splicing or allele) using a *de novo* alternative splice detection pipeline (Liu et al. 2017), a reciprocal self‐BLAST search for all protein sequences was performed to identify putative paralog pairs, which were further filtered out with identities >70% and alignment coverage >50%. The protein sequences of paralog pairs were aligned with MUSCLE, and the protein alignments were back‐translated to coding sequence (CDS) alignments using TreeBest software. The CDS alignments were used to calculate the Ka/Ks values using the CodeML (pairwise mode) of PAML (Yang, 2007).

Lastly, we performed a systematic cross‐species analysis between taimen and seven other salmonid species, including Atlantic salmon (*Salmo salar*) (Lien et al., 2016), rainbow trout (*Oncorhynchus mykiss*) (Berthelot et al., 2014), brown trout (*Salmo trutta*), brook trout (*Salvelinus fontinalis*), and grayling (*Thymallus thymallus*), as well as northern pike (*Esox lucius*) (Rondeau et al., 2014), zebrafish (*Danio rerio*) (Howe et al., 2013), and medaka (*Oryzias latipes*) (Kasahara et al., 2007). The transcript and protein sequences of brown trout, brook trout, and grayling were downloaded from the PhyloFish database (Pasquier et al., 2016). The eggNOG database (Huerta‐Cepas et al., 2016) was used to define the gene family, and eggNOG‐mapper was used to search the fiNOG dataset for orthologous groups (Huerta‐Cepas et al., 2017). To reconstruct the species tree and calculate the divergence time, single‐copy gene families were extracted and the protein sequences were aligned with MUSCLE. The phylogenetic analysis of the superalignments was performed using a maximum‐likelihood algorithm implemented in the PhyML (Guindon et al., 2010) package with the JTT+G+F model calculated using the Smart Model Selection (Lefort, Longueville, & Gascuel, 2017) (SMS, http://www.atgc-montpellier.fr/sms/), and a Bayesian inference method implemented in the MrBayes package (Ronquist & Huelsenbeck, 2003) with the model used to PhyML. The divergence time was calculated using MCMCtree with the approximate likelihood method (Yang & Rannala, 2006). The divergent age between zebrafish and medaka from the TimeTree database (Hedges, Marin, Suleski, Paymer, & Kumar, 2015) was used to calculate a molecular clock date.

### 2.7 Microsatellite marker and SNP discovery

For SSRs, we used modified Sputnik (La Rota, Kantety, Yu, & Sorrells, 2005) to search di‐, tri‐, tetra‐, and pena‐nucleotide unit SSRs, and Primer3 (Untergasser et al., 2012) was then used to design the primers. For the SNPs, we first use Bowtie2 (Langmead & Salzberg, 2012) for mapping clean reads to transcripts and then called SNPs using SAMtools (Li et al., 2009). Raw SNPs with a minimum depth of 10, minimum none reference frequency of 0.1, maximum none reference frequency of 0.9, minor allele frequency of 0.05, and minimum quality of 20 were filtered out using Vcftools (Danecek et al., 2011), and SNPs clustered within 50 bp were also filtered out.

To develop polymorphism microsatellite markers, we synthesized 42 pairs of primers to characterize the polymorphisms. Thirty‐two wild individuals collected from the Hutou section of the Wusuli River (E133°40′17″, N45°58′50″) were used to screen these primers. We used next‐generation sequencing technology to identify alleles and genotypes, and the method is described below (detailed in Appendix S1).

Genomic DNA was extracted from caudal fins and diluted to 250 ng/μL. We used four primers for the PCR assay (Figure S1). The first pair of primers was used to amplify the target amplicon, and an M13 universal primer was concatenated at the 5′ end of the target primers (sequences are listed in Table S2). The second pair of primers were index primers that contained four protective bases, 10 index bases, and M13 universal primer bases. After amplification, the PCR products were quantified with a NanoDrop 8000 system (Thermo Fisher, USA) and pooled at equal amounts to construct sequencing libraries using a TruSeq Custom Amplicon Kit (Illumina, CA, USA). The library was sequenced on a HiSeq 2500 platform in 250‐bp paired‐end mode at the Berry Genomics Co., Ltd. (Beijing, China).

We developed a pipeline to extract genotypes from the sequenced reads (Figure S2). The sequenced raw reads were first processed to trim the sequencing primers and adaptors using Cutadapt software (Martin, 2011), and they were then demultiplexed and classified to each locus and individual using an in‐house program (described in Appendix S1). The cleaned and demultiplexed paired‐end reads were merged using the PEAR program (Zhang, Kobert, Flouri, & Stamatakis, 2014) with the default parameters. We used a modified algorithm in the MEGASAT software (Zhan et al., 2016) to define the genotypes for each locus and individual. Compared with the original version of MEGASAT, we adopted the algorithm from Sputnik software (La Rota et al., 2005) to search the microsatellite repeat array. Each microsatellite repeat array was used as a different locus to avoid the length artifacts contributed by PCR errors and sequence indels of bases between microsatellites if an amplicon contained more than one microsatellite. The genotype decision rule was the same as in the original version. After automatic genotyping, we curated the genotypes manually according to the allele depth. The observed heterozygosity (*H*_o_), expected heterozygosity (*H*_e_), and polymorphism information content were calculated, and the Hardy–Weinberg equilibrium was analyzed (Kuang, Tong, Xu, Yin, & Sun, 2009).

## 3 RESULTS

### 3.1 Transcriptome assembly and assessment

**Figure 2:**
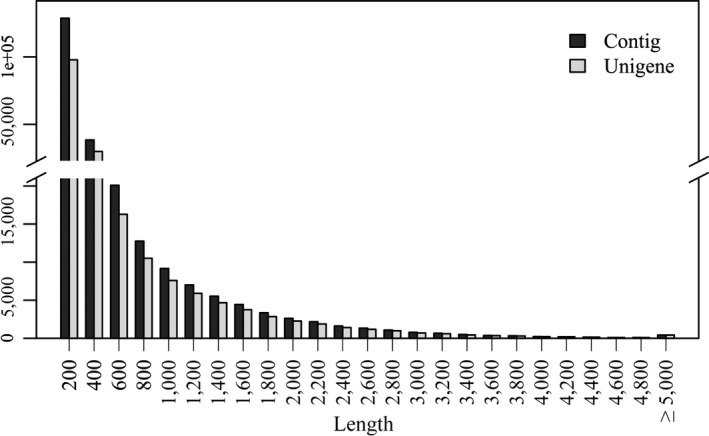
Length distribution of contigs and unigenes

To obtain a whole reference transcriptome, two pooled RNA‐Seq libraries were constructed using RNA samples from 12 organs from 10 individuals. A total of 236.8 M raw reads were generated using the HiSeq 2000 platform in 100PE mode, after trimming the low‐quality bases, 14.15 Giga bases (153 M clean reads) were used to assemble the transcriptome (Table 1). Using the Trinity assembler, 242,069 contigs were obtained with 156.6‐Mb total bases, a 647‐bp average length, and a 943‐bp N50; in addition, 113,359 contigs had a length of more than 400 bp (Figure 2). We performed clustering contigs using TGICL (Pertea et al., 2003), and 190,473 unique clusters were generated with 129.2‐Mb total bases, a 679‐bp average length, and 1,060‐bp N50 size (Table 1, Figure 1). The raw RNA‐Seq reads and assembled transcripts were deposited in the European Nucleotide Archive under the project ID PRJEB19675 and accession numbers HAGJ01000001 to HAGJ01190473 for the assembled transcripts.

**Table 1:** Summary of the reads and assembly transcriptome of taimen

Reads	Pool 1	Pool 2
Raw reads	157,823,830	79,012,698
Clean reads	103,521,538	54,834,108
Total clean bases	14.15 Gb	
Assembly		
Contig numbers	242,069	
Contig bases (bp)	156,662,763	
Contig mean (bp)	647	
Contig N50 (bp)	983	
Number contigs over N50	43,069	
Unigene numbers	190,478	
Unigene bases (bp)	129,286,052	
Unigene mean (bp)	679	
Unigene N50 (bp)	1,060	
Number unigenes over N50	33,431	

To assess the assembly, clean reads were mapped back to the assembled transcripts using Bowtie2, and the results showed that 96.11% of the total clean reads were mapped properly, which meant that 96.11% paired reads were mapped to the same transcript with the appropriate insert size. The full‐length protein‐coding transcripts were examined, and we searched transcripts against several protein databases using Blastx and Blastp. The results showed that 14,666 transcripts represented full‐length proteins, 26,559 transcripts nearly represented the full‐length protein with >80% protein coverage, and 30,284 transcripts covered more than 50% of the protein length (Table 2, Table S1).

**Table 2:** Summary of the taimen transcript annotations

Database/Species	Nucleotide level	Protein level	
	Blastn	Blastp	Blastx
NR		106,289	92,785
Swiss‐Prot		71,407 (4,291)	71,183 (3,614)
NT	78,195		
RefSeq	145,293	86,748 (10,743)	88,846 (8,862)
*Salmo salar*	149,425	85,618 (10,679)	87,878 (8,907)
*Oncorhynchus mykiss*	106,805	82,004 (69)	84,794 (6,651)
*Danio rerio*	15,050	68,592 (6,118)	78,482 (5,142)
*Gasterosteus aculeatus*	21,012	65,566 (5,459)	74,333 (4,694)
*Oryzias latipes*	15,493	63,598 (4,924)	73,633 (4,244)
*Takifugu rubripes*	16,968	64,579 (4,834)	73,148 (4,223)
*Tetraodon nigroviridis*	16,457	62,830 (4,814)	71,355 (4,161)
Total	157,172 (14,666)		

BLAST tools were used for the homology searches with a cutoff *e*‐value of 1e‐10 (nucleotide level) or 1e‐5 (protein level); values in parentheses indicate the number of full‐length transcripts of taimen.

Thirteen mitochondrial protein‐coding genes (NC_016426.1) and 20 mRNA sequences from GenBank were used for the similarity analysis with Blastn to assess the transcript sequence quality (Table S3). All 13 mtDNA protein‐coding genes had hits in the assembly with coverage >96% (11 genes with 100% coverage); 11 sequences matched the assembled transcripts with identities above 99%; and two sequences matched the transcripts with identities of 91%–93%. Among the 20 mRNAs from GenBank, 15 mRNAs were partial CDSs that matched the assembled transcripts with alignment coverage above 87% (13 with coverage > 98%) and identities above 94% (11 with identities > 99%). Except for one complete CDS (JF951962), the other four complete CDSs from GenBank had alignments with identities above 99% and coverage above 73% (KF554113 with coverage 73%, others with coverage above 97%).

Furthermore, completeness was assessed based on the conserved ortholog content. The BUSCO program was used with the Actinopterygii lineage dataset (Simão et al., 2015). In total, 74.7% of the single‐copy orthologs were presented, including 1,371 single‐copy complete orthologs, 1,189 duplicated complete orthologs, and 867 fragmented orthologs (Table S4).

Lastly, gene coverage was assessed by aligning the transcripts to the Atlantic salmon genome and rainbow trout genome using the GMAP program. In total, 9,012 transcripts were classified as chimeric sequences and filtered out for the subsequent analysis. In addition, 155,125 transcripts were mapped to the Atlantic salmon genome with a query coverage above 70% and 105,750 loci were identified based on the alignment overlap, which covered 75.1% (73,436) of the total transcripts in Atlantic salmon (Lien et al., 2016); and 131,887 transcripts were mapped to the rainbow trout genome with a query coverage above 70% and 98,286 loci were identified based on the alignments’ overlap, which covered 57.3% transcripts (26,709) in rainbow trout (Berthelot et al., 2014).

### 3.2 Functional annotation

We used TransDecoder to predict the ORFs and translation protein sequences. The Swiss‐Prot and Pfam databases were used to identify the ORFs, and 107,242 transcripts (56.3% of total transcripts) were used to successfully predict ORFs with an average size of 196 amino acids and a N50 size of 314 amino acids. As the complement to TransDecoder, GeneMarkS‐T program (Tang et al., 2015) was used to predict ORFs, and 63,831 ORFs were obtained, after removing redundant ORFs determined by Cd‐hit package (Li & Godzik, 2006), 2,240 ORFs were added to TransDecoder prediction set, and finally 109,482 transcripts were successfully predicted ORFs. We then used several complementary routes to annotate the transcript sequences. First, we performed BLAST searches of taimen transcripts against several databases and found that 157,172 (82.5%) transcripts had significant sequence homology to the databases, and most (96.3%) were annotated with reference transcripts and proteins of Atlantic salmon (Lien et al., 2016) (Table 1). A homology search of the Pfam databases showed that 52,762 transcripts had at least one significant hit, and 79,800 transcripts were annotated using Interproscan (Jones et al., 2014).

**Figure 3:**
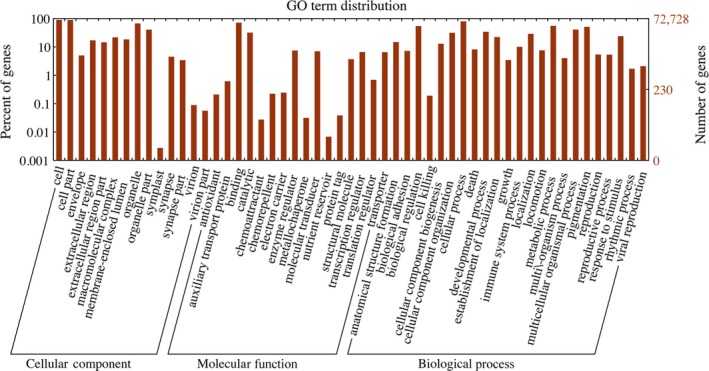
GO term distribution of taimen transcripts

Second, GO annotation was performed. The sequences with significant hits in the Uniprot database or Pfam database were assigned GO terms using the Trinotate package, and the GO terms were assigned using Interproscan (Jones et al., 2014). Among the total transcripts, 72,728 transcripts were assigned to 15,107 GO terms, including 10,185 biological process terms, 1,429 cellular component terms, and 3,493 molecular function terms (Figure 3). Cytoplasm (GO:0005737), nucleus (GO:0005634), integral component of membrane (GO:0016021), and plasma membrane (GO:0005886) were most abundant and accounted for 29.7%, 28.4%, 19.1%, and 17.1% of the total assigned transcripts, respectively.

A KEGG pathway analysis was performed using GhostKOALA (Kanehisa et al., 2016). A total of 51,698 transcripts were assigned to 8,052 KEGG ortholog groups, and among these transcripts, 28,886 were classified into 385 KEGG pathways, including metabolism (6,224 transcripts in 139 pathways), genetic information process (4,966 transcripts in 22 pathways), environmental information process (10,743 transcripts in 38 pathways), cellular processes (8,716 transcripts in 25 pathways), organic systems (11,577 transcripts in 81 pathways), and others (11,543 transcripts in 80 pathways) (Table S5). The top 10 metabolism pathways with the most transcripts were purine metabolism (710), lysine degradation (496), inositol phosphate metabolism (469), glycerophospholipid metabolism (398), pyrimidine metabolism (353), oxidative phosphorylation (352), glycerolipid metabolism (264), glycolysis/gluconeogenesis (255), N‐glycan biosynthesis (217), and amino sugar and nucleotide sugar metabolism (202).

**Figure 4:**
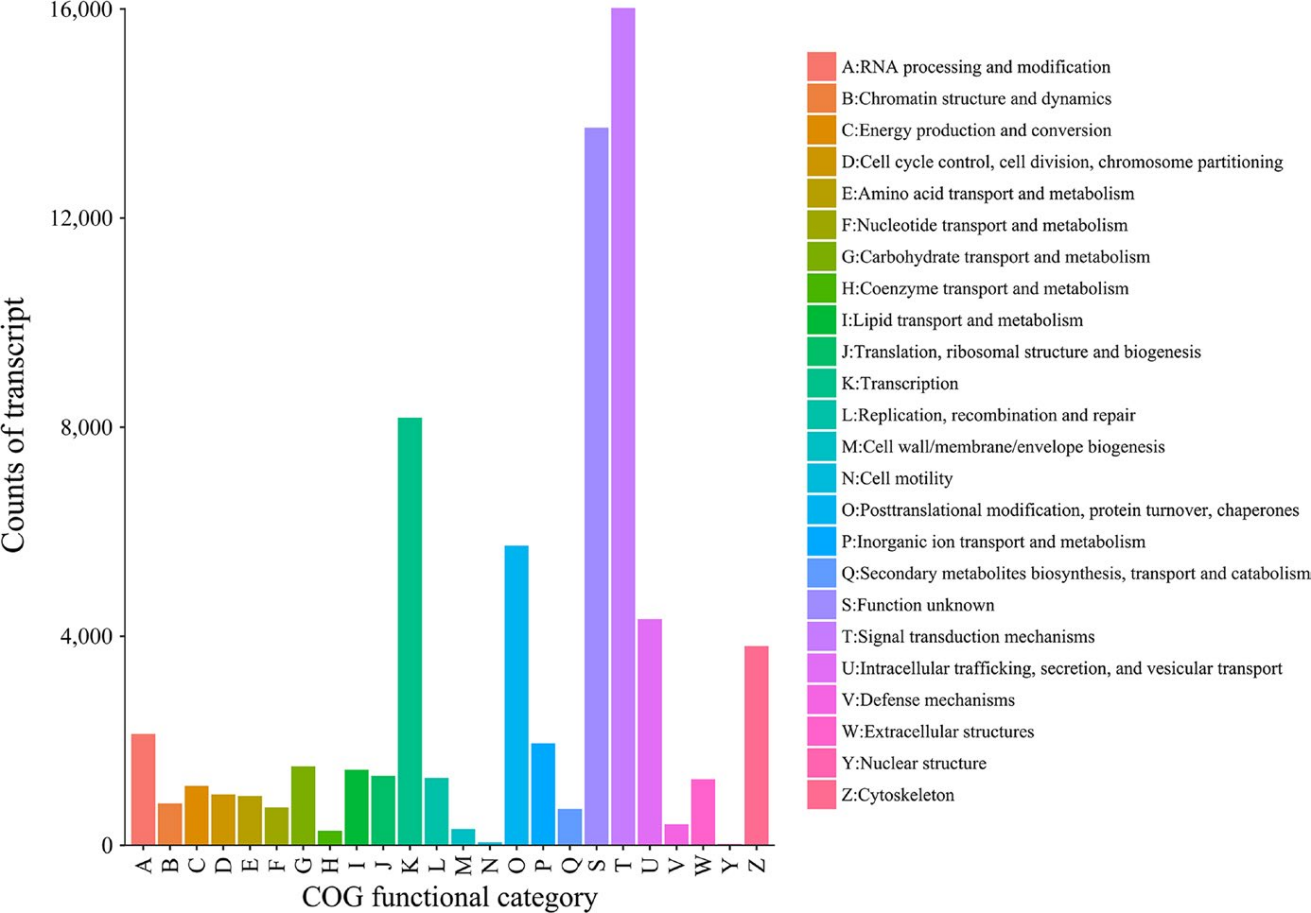
Distribution of taimen transcripts classified by COG function

Meanwhile, the COG functional category annotation using eggNOG‐mapper (Huerta‐Cepas et al., 2017) yielded good results and a total of 72,605 putative proteins. These COG‐annotated putative proteins were successfully classified into 125 COG functional categories, and most were homologous to 32 molecular families, including signal transduction mechanisms, transcription, post‐translational modification, cytoskeleton, and intracellular trafficking and secretion (Figure 4).

### 3.3 Homology analysis

**Figure 5:**
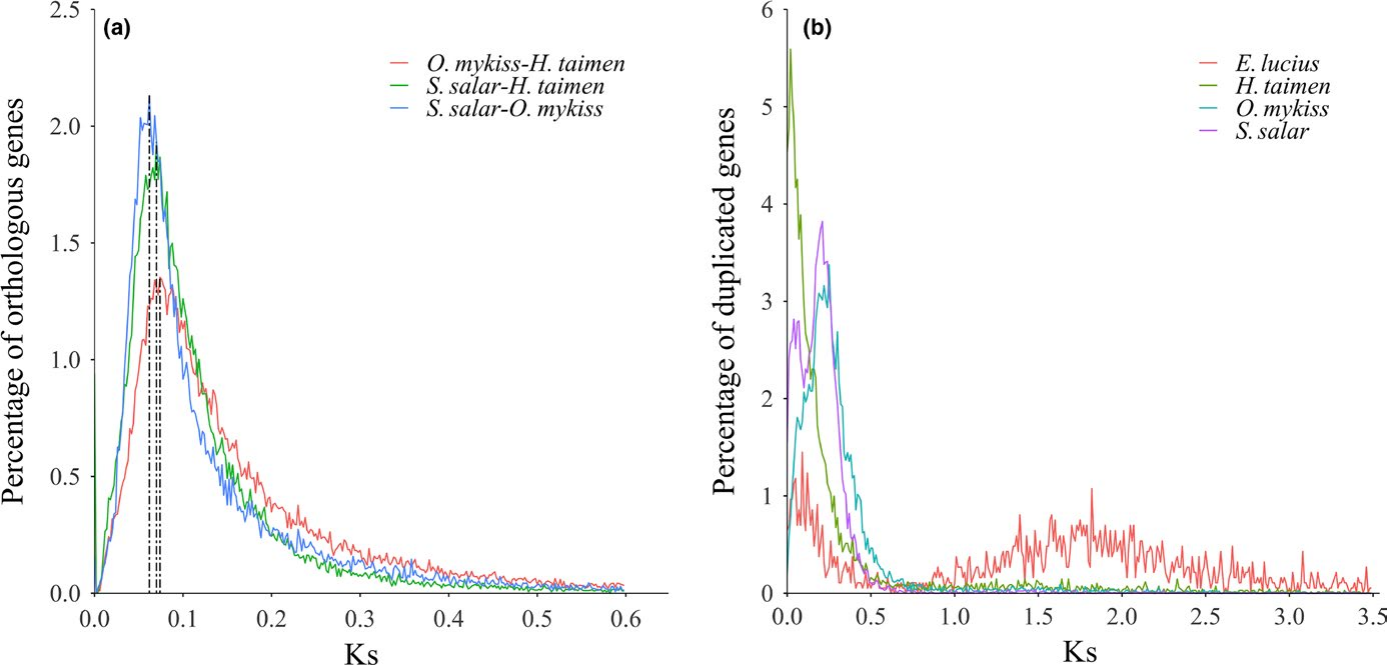
Homologous analysis among taimen and other three teleosts. (a, b) Distribution of the synonymous substitution rates (Ks) of homologous genes for interspecies (a) and intraspecies and (b) a comparison of taimen, rainbow trout, Atlantic salmon, and northern pike. We used homologous pairs with Ks values <0.6 (a) and <3.5 (b) to plot the Ks distribution curves. Vertical dashed lines in (a) marked the Ks values (0.062, 0.070, 0.074, respectively) of peaks

Among salmonids, many species transcriptomes and the entire genome of Atlantic salmon and rainbow trout have been published, and these data were helpful in the comparative analysis between taimen and other salmonids. We first searched the orthologs between taimen and Atlantic salmon using the CRB‐BLAST algorithm (Aubry et al., 2014) and found that for 53,707 orthologous pairs between taimen and Atlantic salmon, the Ks distribution of these orthologous pairs showed that 97.6% pairs had Ks values <0.6 and a median value of 0.09. The Ks distribution profile showed a peak *K*s value of 0.070 (Figure 5a). The orthology analysis between taimen and rainbow trout showed 53,738 orthologous pairs. The Ks distribution of these orthologous pairs showed a Ks peak at 0.074 (Figure 5a), with a median value of 0.119%, and 91.3% of pairs had Ks values below 0.6. We also searched for orthologs between Atlantic salmon and rainbow trout and found 34,622 orthologous pairs; among these, 96.9% of the pairs had Ks values below 0.6, and the Ks distribution showed that a peak occurred at a Ks value of 0.062 (Figure 5a), with a median value of 0.087. Considering the synonymous substitution rate of 3.1 × 10^−9^ substitutions/synonymous site per year (Crête‐Lafrenière, Weir, & Bernatchez, 2012), speciation between Atlantic salmon and rainbow trout was estimated to have occurred 20–28 MYA, and the event in which the *Hucho* genus diverged from *Oncorhynchus* and *Salmo* occurred at approximately 22 MY–38 MY.

We then searched intraspecies duplicated genes across taimen, Atlantic salmon, rainbow trout, and northern pike using a RBH approach. Northern pike, whose genome underwent the teleost‐specific third round whole‐genome duplication (WGD) (hereafter, termed Ts3R), is a member of the closest diploid sister group to salmonids, and we used it as a reference to compare the divergence of the duplicated genes of salmonids. The ancestral genome of salmonids reportedly underwent an additional round for the whole genome, that is, salmonid‐specific fourth WGD (hereafter, termed Ss4R). We found 2,256, 12,524, 8,987, and 8,966 pairs of duplicated genes for northern pike, Atlantic salmon, rainbow trout, and taimen, respectively. The Ks distribution profiles are shown in Figure 5b, which indicates that most pairs of duplicated genes (82.5%) of northern pike had Ks values <3.5, and the Ks were mainly distributed from 0.75 to 3.5, with a major peak at around 1.75. The Ks distribution profile was similar to that of other Ts3R teleosts (Berthelot et al., 2014; Jaillon et al., 2004). Although the three salmonids had the most pairs of duplicated genes with Ks values below 0.5 (89.7%, 88.7%, and 86.9%), the Ks distribution profiles were different. Atlantic salmon had a major peak at a Ks value of approximately 0.21 and a secondary peak at a Ks value of approximately 0.06, whereas rainbow trout and taimen only showed major peaks at Ks values of approximately 0.25 and 0.02, respectively.

### 3.4 Gene family definition and phylogenetic analysis

**Figure 6:**
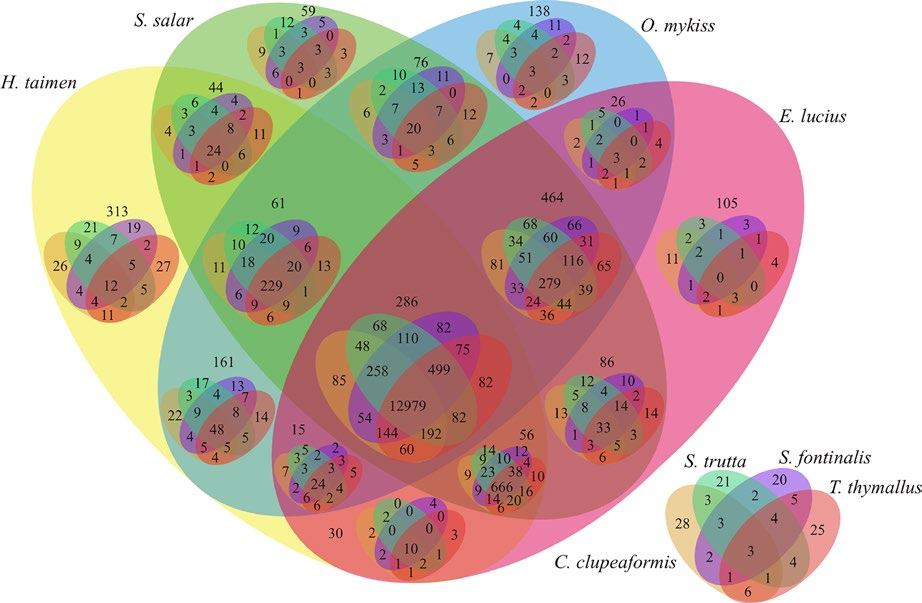
Net Venn diagram of gene families among eight species. Net Venn diagram was drawn using VennPainter (Lin et al., 2016); numbers in overlapped ellipse represented the number of gene families shared by species, numbers in nonoverlapped ellipse represented the number of unique gene families of a species; 12,979 families were shared by the eight species; 313 families occurred only in taimen

We used 11 teleosts (eight species in Salmonidae, northern pike, medaka, and zebrafish) for a systematic comparative analysis and classified the genes according to their similarity. A total of 17,533 orthologous groups were classified for taimen, including 72,605 putative proteins. A total of 12,979 groups were shared by eight salmonids, and 313 groups containing 491 transcripts occurred only in taimen (Figure 6). These groups mainly involved signal transduction mechanisms, post‐translational modification, transcription, intracellular trafficking, and cytoskeleton (Figure S3). These groups were also enriched in the focal adhesion pathway (KEGG, dre04510, *p* < .05) and 6 GO terms (*p* < .05), including GTP binding (GO:0005525), protein delipidation (GO:0051697), transcription regulatory region DNA binding (GO:0044212), small GTPase‐mediated signal transduction (GO:0007264), C‐terminal protein lipidation (GO:0006501), and protein targeting to membrane (GO:0006612).

**Figure 7:**
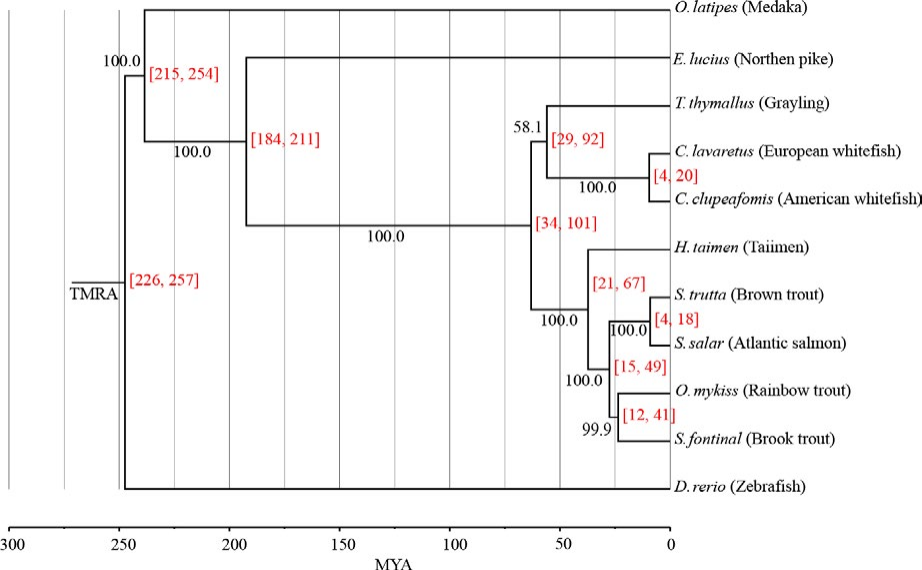
Phylogenetic tree of salmonids and other related teleosts. Single‐copy orthologs were used to reconstruct the phylogenetic tree with PhyML and MrBayes, and the model JTT+I+G+F was used; the topologies of phylogenetic trees inferred by PhyML and MrBayes were consistent; numbers with a black color on the branch are the bootstrap values, and divergent ages were calculated with MCMCtree program in PAML package. The 95% confidence interval for age is shown in square brackets in red. Gray vertical lines are reference lines of ages. TMRA, the most recent ancestor

We then identified 134 single‐copy orthologs that were conserved among all 11 species and used the multiple alignments of these single‐copy orthologs to reconstruct species phylogenies with a maximum‐likelihood approach (PhyML) and a Bayesian inference method (MrBayes). The topologies of phylogenetic trees analyzed by PhyML and MrBayes were consistent (Figure S4). The species phylogeny and divergent age showed that Salmonidae originated 63 MYA with a 95% confidence interval (CI) of 34–101 MYA. The speciation event of taimen was estimated to have occurred at 37 MYA with 95% CI of 21–67 MYA (Figure 7).

### 3.5 Positive selected genes

Taimen are restricted to freshwater ecosystems, whereas Atlantic salmon and rainbow trout live in seawater. These different ecological habitats might generate different selective pressures between the two types of salmonids. Using Atlantic salmon as the reference, we characterized 1,128 putative positive selection transcripts (Ka/Ks > 1) and then used the F3 × 4 model of codon frequencies and sites models of M7 and M8 to test the significance. We identified 250 transcripts that were positively selected (FDR < 0.05), and 128 transcripts were annotated by zebrafish proteins (Table S6). We used 128 transcripts for an enrichment analysis with DAVID Bioinformatic Resources (Huang, Sherman, & Lempicki, 2009) and found that three pathways/terms were enriched (Benjamini‐adjusted *p* value < .05): cytokine–cytokine receptor interaction (KEGG pathway dre04060), integral component of membrane (GO:0016021), and membrane (GO:0016020) (Table 3, Table S6). To further examine whether these transcripts were positively selected in the taimen lineage, we performed a branch‐site model test using orthologs from Atlantic salmon and rainbow trout as the background and found 67 of 128 taimen transcripts were significantly positively selected (FDR < 0.05) (Table S7). The enrichment analysis showed that four pathways/terms were enriched (Benjamini‐adjusted *p* value < .05), and they involved cytokine–cytokine receptor interactions (KEGG dre04060), immune response (GO:0006955), chemokine activity (GO:0008009), and cytokine activity (GO:0005125) (Table 3).

**Table 3:** Enrichment analysis of the positive selection genes of taimen

Model	Category	Term	Description	Count	*p* Value
Sites (M7, M8)	KEGG Pathway	dre04060	Cytokine–cytokine receptor interaction	17	.0012
	Gene Ontology	GO:0016021	Integral component of membrane	93	.0113
	Gene Ontology	GO:0016020	Membrane	95	.0428
Branch‐sites	KEGG Pathway	dre04060	Cytokine–cytokine receptor interaction	11	9.60E‐04
	Gene Ontology	GO:0008009	Chemokine activity	5	.0080
	Gene Ontology	GO:0006955	Immune response	11	.0138
	Gene Ontology	GO:0005125	Cytokine activity	8	.0140

Atlantic salmon was used as a reference to perform the site model tests, and orthologs of Atlantic salmon and rainbow trout were used as the background for branch‐site model tests. Count represents the number of transcripts involving a specific pathway. The *p* value was adjusted via the Benjamini method.

### 3.6 Microsatellite marker and SNP discovery

Single‐nucleotide polymorphism markers were called using SAMtools (Li et al., 2009). In total, 396,424 SNPs were called, and after filtering out the low‐quality SNPs and clustering SNPs within a window size of 50 bp, 68,533 SNPs remained. Among them, transition SNPs accounted for 62.5% of the total; 42,017 SNPs located in a CDS region accounted for 61.3% of the total; and 28,387 synonymous variants accounted for 41.4% of total SNPs (Table 4).

**Table 4:** Summary of the SNPs detected using SAMtools in taimen

Type	Count
A‐C/C‐A	7,469
A‐G/G‐A	21,680
A‐T/T‐A	6,166
C‐G/G‐C	4,441
C‐T/T‐C	21,158
G‐T/T‐G	7,555
Transition	42,838
Transversion	25,631
Coding SNP	42,017
Synonymous SNP	28,387

Microsatellite markers are a useful tool for genetic analysis in nonmodel species. We used modified Sputnik software to search di‐, tri‐, tetra‐, pena‐, and hex‐nucleotide motif SSRs and obtained 17,841 SSRs with at least six repeats in 14,645 sequences. There were 62 types of repeat units in the SSRs, and among them, dinucleotide unit repeats (82.9%) were more abundant than the others. The AC unit was most abundant and accounted for 52.7% of the microsatellites (Figure S5). In terms of repeat time, unit AC had the maximum repeats of 107, and most units were repeated <30 times (Figure S6). Primers were designed using the Primer3 package, and 6,745 pairs of primers were designed successfully. After aligning the amplicon sequences to the Atlantic salmon genome with BLAT, 2,361 pairs of primers were classified as good primers because their amplicons were located in the genome of Atlantic salmon with identities >70% and spanned distances close to the length of the amplicons.

We synthesized 42 pairs of primers to characterize the polymorphisms. Among them, four pairs of primers failed to sequence because of the few reads obtained (Table 5). We obtained a total of 4.2 M reads, and after trimming the adapter and sequencing primers, we assigned 3.4 M reads to 38 loci and demultiplexed 1.7 M reads to 32 individuals. The mean depth of each locus ranged from 258 to 2,974 (Figure S7), and the mean depth of each sample ranged from 895 to 1,764 (Figure S8). The reads were deposited in the European Nucleotide Archive under the project ID PRJEB19675. Using the Sputnik algorithm, we successfully identified 43 microsatellites. Five amplicons contained two microsatellites, and among them, 24 polymorphism loci were characterized with alleles ranging from 2 to 8 and a mean number of 3.4167. Ten loci contained two alleles, and six loci were highly polymorphic with at least five alleles (Table 5).

**Table 5:** Polymorphism screen results of 42 pairs of primers

Primers	Repeat unit	Alleles	*A*_o_	*A*_e_	*H*_o_	*H*_e_	PIC	HWE
HtaC1002	GT	20	Monomorphic					
	GT	33	Monomorphic					
HtaC1003	GT	19, 23, 25, 27, 29, 31, 35, 37	8	5.83	0.88	0.83	0.81	0.05
HtaC1004	CT	31	Monomorphic					
HtaC1005	AAAAC	14	Monomorphic					
	CT	14, 16, 19, 31	4	2.88	1.00	0.65	0.59	0.00
HtaC1006	Failed to sequence							
HtaC1008	GT	44, 48	2	1.79	0.41	0.44	0.34	0.69
HtaC1009	CT	21	Monomorphic					
HtaC1010	CT	27, 31	2	1.56	0.47	0.36	0.29	0.16
HtaC1011	AC	18, 20	2	1.06	0.06	0.06	0.06	1.00
HtaC1012	AC	18	Monomorphic					
HtaC1013	ATT	13	Monomorphic					
HtaC1015	AG	17, 19, 21	3	2.60	0.69	0.62	0.54	0.56
HtaC1016	AAC	12, 15, 18, 21, 24, 27	6	3.07	0.50	0.67	0.61	0.05
HtaC1017	GT	19	Monomorphic					
HtaC1019	GT	13, 19, 21	3	1.62	0.25	0.38	0.32	0.06
HtaC1020	GT	13	Monomorphic					
	GT	21	Monomorphic					
HtaC1024	AG	13	Monomorphic					
HtaC1025	GAT	16, 19	2	2.00	0.59	0.50	0.37	0.48
HtaC1026	GT	18, 20	2	1.98	0.34	0.50	0.37	0.09
HtaC1027	AC	13	Monomorphic					
HtaC1028	AT	13, 14, 15	3	2.09	0.41	0.52	0.41	0.01
HtaC1029	AC	13, 17	2	1.60	0.44	0.38	0.30	0.64
HtaC1030	Failed to sequence							
HtaC1031	AAC	16, 19	2	1.60	0.38	0.38	0.30	1.00
HtaC1032	GT	18, 20	2	1.52	0.25	0.34	0.28	0.13
HtaC1033	Failed to sequence							
HtaC1034	ATT	15, 18, 21	3	2.02	0.52	0.51	0.44	1.00
HtaC1035	GATCT	16	Monomorphic					
	CT	12, 14	2	1.48	0.41	0.32	0.27	0.30
HtaC1036	Failed to sequence							
HtaC1038	AC	32	Monomorphic					
HtaC1041	AC	34	Monomorphic					
HtaC1042	AC	23, 25, 27, 29, 31	5	3.23	0.93	0.69	0.65	0.15
HtaC1043	AC	14	Monomorphic					
HtaC1044	GTT	16	Monomorphic					
HtaC1045	GT	16, 18, 20, 22, 28, 30, 32	7	3.17	0.55	0.68	0.64	0.01
HtaC1046	AC	18, 20, 22, 24, 26, 28	6	3.84	0.84	0.74	0.70	0.67
HtaC1047	GT	20, 22	2	1.10	0.09	0.09	0.09	1.00
HtaC1048	AC	26, 30, 32	3	1.37	0.25	0.27	0.25	0.57
HtaC1049	AAAT	32	Monomorphic					
	AT	15, 17, 19	3	2.73	0.53	0.63	0.56	0.01
HtaC1050	GT	19	Monomorphic					
HtaC1051	AC	15, 17, 19, 21, 23, 25	6	3.62	0.72	0.72	0.69	0.70
HtaC1053	AT	18	Monomorphic					
Mean			3.42	2.28	0.48	0.47	0.41	

A locus may contain more than one microsatellite repeat array, and alleles are represented by the length of the repeat array.

*A*_o_, observed allele count; *A*_e_, expected allele count; *H*_o_, observed heterozygosity; *H*_e_, expected heterozygosity; PIC, polymorphism information content; HWE, *p* value of Hardy–Weinberg equilibrium according to the Fisher exact test.

## 4 DISCUSSION

Taimen is an ecologically and economically important species, and the development of genetic tools, such as genetic markers, is useful for conservation biology, aquaculture, and genetic breeding. In this study, we performed RNA sequencing using two pooled‐tissue RNA libraries and assembled a high‐quality transcriptome for taimen, and it was assessed with five stringent criteria. In the assembly, 82% of transcripts were annotated by several databases, and 14,666 of the transcripts contained a full coding region (Table 1). In all, 41,971 transcripts had the best protein hits, which presented coverage of >50% (Table S1). The assembly covered 75% of the transcripts of Atlantic salmon and 57.3% of the protein‐coding genes of rainbow trout. Based on the conserved ortholog content analysis, the taimen transcripts covered 74.7% of the Actinopterygii lineage single‐copy orthologs, and 55.8% of the Actinopterygii lineage single‐copy orthologs were completed covered by 4,218 taimen transcripts. The pooled‐tissue RNA sequencing has been demonstrated to be an effective approach for obtaining the maximum number of independent genes (Smith et al., 2001; Wang, Li, Zhang, & Sun, 2012). Francis et al. (2013) studied the effect of sequencing depth for *de novo* transcriptome assembly in nonmodel species and found that 20 million reads for tissue samples and 30 million reads for whole animals were sufficient to assemble the representative transcriptome. We obtained 157 M clean reads (14 Gb) in total, with the 3 Gb genome of Atlantic salmon used as a reference, and assuming that 1% of the genome encodes mRNAs (Wang et al., 2012), we sequenced the taimen transcriptome at a depth of approximately 450×, which satisfied the demand for the *de novo* assembly of the transcriptome.

In this study, we estimated the speciation age and molecular divergence rate of taimen. To validate our approach for estimating speciation age, we compared the calculated speciation age and the molecular divergence rate of *Salmo* using our RNA‐seq data with published results. Previous studies shown that the salmonid ancestral genome underwent the fourth WGD at ~79 MYA, and *Salmo* diverged from *Oncorhynchus* at ~21 MYA (Berthelot et al., 2014; Lien et al., 2016; Macqueen & Johnston, 2014; Shedko, Miroshnichenko, & Nemkova, 2013). In our study, we estimated that *Salmo* diverged from *Oncorhynchus* at 27 MYA with a 95% CI of 15–49 MYA, which is similar to the results of previous studies. Using the median value of 0.087 of the pairwise Ks between Atlantic salmon and rainbow trout, the estimated synonymous substitution rate was 3.2 × 10^−9^ substitutions/synonymous site per year with a 95% CI of 1.8 × 10^−9^–5.8 × 10^−9^, which is also similar to that of Crête‐Lafrenière, Weir, and Bernatchez (2012). These similar results supported our strategies used in this study. Based on the molecular divergence rate of 3.1 × 10^−9^, together with the resulted pairwise Ks values of the interspecies orthologs, the taimen speciation age was estimated to be 22–38 MYA after considering estimation from phylogenetic divergence analysis (Figures 5a and 7).

The Ks value of paralogous genes can be used to reveal WGD events (Wang et al., 2012; Xu et al., 2014). Northern pike is a species of teleost whose genome underwent third round WGD. Our analysis successfully demonstrated that the Ks distribution profile of pairwise paralogous genes of Northern pike presented a classic Ts3R distribution curve (Figure 5b) (Berthelot et al., 2014; Jaillon et al., 2004). Atlantic salmon and rainbow trout had major Ks peaks at values of approximately 0.21 and 0.25, respectively (Figure 5b), and based on these peak Ks values and the molecular divergence rate 3.1 × 10^−9^, we estimated that the ancestral WGD event of salmonids occurred at 67–80 MYA (Figure 5b), which is consistent with the results of previous studies (Berthelot et al., 2014; Crête‐Lafrenière et al., 2012; Lien et al., 2016; Macqueen & Johnston, 2014; Shedko et al., 2013).

Although the Ks distribution showed major peaks at Ks values of approximately 0.21 and 0.25 for Atlantic salmon and rainbow trout, respectively, Atlantic salmon had a minor peak at Ks 0.06 (Figure 5b), indicating different divergent patterns for duplicate genes in Atlantic salmon and rainbow trout. The observed genome rediploidization showed that different rediploidization patterns and duplicate retention patterns and mechanisms occurred in Atlantic salmon and rainbow trout. At least 50%–60% Ss4R gene duplicates were retained in the salmon genome, whereas only 48% Ss4R gene duplicates were retained in the rainbow trout genome. Rediploidization analyses showed that up to 25% of the salmon genes showed delayed rediploidization after chromosome arrangement (Berthelot et al., 2014; Lien et al., 2016). Thus, more duplicated genes were observed in salmon than rainbow trout, and the divergence of gene duplicates in salmon was slower than that in rainbow trout. The Ks distribution profiles of intraspecies homologous genes provided more evidence that the evolutionary progress of Atlantic salmon varied from that of rainbow trout. The Ks distribution of pairwise paralogous genes in taimen showed a distinct profile and a major peak was observed at Ks 0.02 (Figure 5b), which might have been caused by incomplete transcriptomes and incomplete coding regions. Although the taimen transcriptome covered approximately 70% of the genes of Atlantic salmon and 50% of the genes of rainbow trout, all pairs of gene duplicates are difficult to obtain. Additionally, the partial coding region might lead to underestimates of the pairwise Ks of gene duplicates within taimen. We obtained 30,284 transcripts that presented protein coverage of more than 50%, and this value might be insufficient to detect all substitute sites.

Salmonids contain two major ecological types: seawater and freshwater. Taimen is a strictly freshwater species, whereas rainbow trout and Atlantic salmon are seawater species. Compared with the number in Atlantic salmon and rainbow trout, we identified 128 positive selected genes for taimen (Table S6) that were mainly involved in immune‐related pathways through enrichment analysis, which suggests that some immune‐related genes in taimen may have undergone strong positive selection. This finding implies that taimen experienced serious challenges based on the environment and disease exposure. The gene family analysis supported this fact, compared to Atlantic salmon and rainbow trout, taimen presented 529 unique families (Figure 6) that were also enriched in GTP binding (GO:0005525, *p* < .01) and small GTPase‐mediated signal transduction (GO:0007264, *p* < .01), which plays an important role in the immune system (Cantrell, 2002). The 529 unique gene families of taimen included genes of Ras super gene family, such as Rap1a, Rab5a, Rab9a, and Rab39b, these genes were also demonstrated that they play cardinal roles in immunity and inflammation (Johnson & Chen, 2012). These results suggest that taimen might have more immune‐related genes than Atlantic salmon and rainbow trout. Whether immune‐related gene enrichment and positive selection are general characteristics for freshwater salmonids has not been clarified.

In this study, we also developed a pipeline for screening polymorphic microsatellite markers that can be used to genotype samples. Microsatellite markers are highly polymorphic, and they are useful for studying population genetics, performing parentage analyses and quantitative trait locus analyses and constructing linkage maps; however, this is a time consuming and costly method of characterizing polymorphism markers. The pipeline we developed is a time‐ and cost‐efficient way to development microsatellite markers. It first added index bases for samples via four PCR primers (Figure S1). The PCR products of all loci and samples were pooled for next‐generation sequencing to minimize costs. The data analysis pipeline contained two main self‐developed programs: The first one demultiplexed the reads to each locus and sample with the sequences of the primer loci and index bases of samples added by PCR (Figure S1), and the second one identified alleles via a modified algorithm of MEGASAT (Zhan et al., 2016). The program is distinguished from previous software (Suez et al., 2015; Zhan et al., 2016) because it adopts the Sputnik algorithm (La Rota et al., 2005) to search the microsatellite repeat array. This characteristic enabled the program to function without background information, such as microsatellite sequences and repeat array flank sequences, and only primer sequences are needed to classify reads to loci and index bases for demultiplexing the reads to samples. The program treated each microsatellite motif as a different locus if an amplicon contained more than one microsatellite. The polymorphism was only identified by the length of the repeat array, thereby eliminating length artifacts contributed by PCR errors and sequence indels of the bases flanking microsatellites. We used the pipeline to characterize 24 polymorphism microsatellite markers from the transcriptome, and these markers could be applied to population or conservation genetics after careful test of their neutrality. The SNPs identified from these data should also be tested neutrality before their application into population genetics.

## DATA ACCESSIBILITY

Raw data were deposited in the European Nucleotide Archive under the project ID PRJEB19675. Sequence assemblies (FASTA) were deposited in the European Nucleotide Archive with accession numbers HAGJ01000001 to HAGJ01190473. Annotations (including Trinotate, Interproscan, eggNog‐mapper, KEGG pathway, and Gene Ontology annotation) and programs for characterization polymorphic microsatellite markers are deposited to Dryad Digital Repository with link https://doi.org/10.5061/dryad.9gd3n

## CONFLICT OF INTEREST

None declared.

## AUTHOR CONTRIBUTIONS

Y.K., J.Y. and G.T. conceived the study; Y.K. and G.T. wrote the manuscript; W.X., Y.Z. and Q.Z. were involved in sample collection and RNA extraction; Y.K. and G.T. analyzed the data; and Y.K. developed the microsatellite pipeline and programs. All authors revised the manuscript and declare no competing financial interests.
