# Supplementary material for: De novo assembly and characterization of the Hucho taimen transcriptome
Source: Ecol Evol. 2017 Dec 21;8(2):1271–85. doi: 10.1002/ece3.3735 (PMC5773338; doi:10.1002/ece3.3735)
Supplement: Supplementary file 1 [file ECE3-8-1271-s001.doc]

**Supplementary Information**

*De novo* assembly and characterization of the Hucho taimen transcriptome

Guang-Xiang Tong, Wei Xu, Yong-quan Zhang, Qing-Yu Zhang, Jia-Sheng Yin*, You-Yi Kuang*

Heilongjiang River Fisheries Research Institute of Chinese Academy of Fishery Sciences, No. 232, Hesong Street, Daoli District, Harbin 150070, Heilongjiang Province, China.

* To whom correspondence should be addressed. Jia-Sheng Yin, E-Mail: [yinjiasheng@hrfri.ac.cn](mailto:yinjiasheng@hrfri.ac.cn); Tel: +86-451-87930960; Fax: +86-451-84604803. You-Yi Kuang, E-Mail: [kuangyouyi@hrfri.ac.cn](mailto:kuangyouyi@hrfri.ac.cn); Tel: +86-451-87930951; Fax: +86-451-84604803.

**Appendix S1.**

**Method for characterizing microsatellite polymorphisms**

Genomic DNA was extracted from caudal fins using phenol-chloroform protocol and diluted to 250 ng/μL. We used four primers to perform the PCR assays (Supplementary Figure S1); the first pair of primers were target primers for amplifying the target amplicon and included a M13 universal primer (M13 forward primer: TGTAAAACGACGGCCAGT, M13 reverse primer: CAGGAAACAGCTATGACC), which was concatenated at the 5’end of the target primers; and the second pair of primers were index primers, which contained 4 protective bases, 10 index bases and a M13 universal primer bases. A touch-down PCR program was used to amplify the product as follows: initial denaturing at 95℃ for 3 min; 10 cycles of 95℃ 30 s, annealing for 30 s, and 72℃ for 30 s; a decrease in the annealing temperature by 1℃ per cycle from 65℃ to 55 ℃; 20-25 cycles of 95℃ for 30s, 58℃ for 30s, and 72℃ for 30s; a final extension at 72℃ for 5 min; and then storage at 4℃. The PCR assay was performed with a 15 μL volume that contained 7.5 μL DreamTaq™ Hot-Start PCR master mix (Thermo Fisher Scientific Inc., CA, USA), 25 ng DNA, 1 μM of each target primer and 5 μM of each index primer. After amplification, the PCR products were quantified with a NanoDrop 8000 and pooled with equal amounts to construct the sequencing library using a TruSeq Custom Amplicon Kit (Illumina, USA). The library was sequenced on a HiSeq 2500 platform in 250 bp paired-end mode at Berry Genomics Co., Ltd (Beijing, China).

The sequenced raw reads analysis workflow is illustrated in Supplementary Figure S2 and is briefly described as following. The raw reads were first processed to trim the sequencing primers and adaptors using Cutadapt software (Martin, 2011) and then demultiplexed into each loci(PCR amplicons) and samples according to the primer and index sequences using an in-house program (DeMultiIndex) that contains two core modules, i.e. Locus assignment module and Index assignment module. We used Needleman-Wunsch global alignment algorithm(hereafter referred to as “NW”) to search target primer sequences and index bases in these modules, the scoring parameters for NW were set as match 1, mismatch -2, gap open -4 and gap extension -1. In the Locus assignment module, we identified target primer sequences to assign loci using maximum gap 2 and minimum similarity 0.85, while in the Index bases assignment module, we first searched the universal primers that were used to concatenate target primers and index bases(Supplementary Figure S1) using NW with the parameters used in the Locus assignment module. We then extracted neighboring sequences 5’ to the universal primers, the length of extracted sequences equaled to the length of index sequences plus 1, we compared the index sequences to the extracted sequences using NW with parameters of maximum gap 1 and minimum similarity 0.95 to identify individual indexes and assign reads to individual samples. After reads successfully assigned to loci, the universal primers and index sequences were removed

Thirdly, the trimmed and demultiplexed paired-end reads were merged using the PEAR program (Zhang, Kobert, Flouri, & Stamatakis, 2014) with default parameters. Finally, we used a modified algorithm using MEGASAT software (Zhan et al., 2016) to define the genotypes for each loci and individual. For comparisons with the original version of MEGASAT, we used the Sputnik software algorithm (La Rota, Kantety, Yu, & Sorrells, 2005) to search the microsatellite repeat array (MRA) and extract flank sequences. If an amplicon contained more than one MRA, each MRA was treated as a different locus to avoid the contribution of length artefacts by PCR errors and sequence indels between MRAs. To count the depth of each MRA, we used the Needleman-Wunsch alignment algorithm with a minimum similarity of 0.85 and a maximum gap of 2 to search the flank sequences of MRA against all reads. The genotype decision rule was the same as the original version.

**Supplementary Tables**

**Supplementary Table S1. Distribution of coverage length for the top matching database entries by Blastx with an e-**value cutoff of 1e-5.

| Database/Species | 100% coverage | | 80% coverage | | 50% coverage | | 10% coverage | | Total | |
| --- | --- | --- | --- | --- | --- | --- | --- | --- | --- | --- |
|  | Query | Target | Query | Target | Query | Target | Query | Target | Query | Target |
| Swiss-Prot | 3,614 | 1,862 | 10,076 | 5,383 | 9,901 | 5,786 | 33,006 | 15,575 | 56,597 | 22,332 |
| RefSeq | 8,862 | 5,921 | 8,925 | 6,231 | 11,311 | 8,720 | 43,059 | 26,301 | 72,157 | 40,137 |
| *Salmo salar* | 8,907 | 5,864 | 8,773 | 5,979 | 10,557 | 7,991 | 42,202 | 24,220 | 70,439 | 36,318 |
| *Oncorhynchus mykiss* | 6,651 | 3,889 | 12,077 | 6,961 | 13,801 | 8,954 | 41,281 | 18,860 | 73,810 | 38,695 |
| *Danio rerio* | 5,142 | 2,676 | 11,275 | 6,017 | 10,976 | 6,795 | 36,151 | 15,343 | 63,544 | 22,521 |
| *Gasterosteus aculeatus* | 4,694 | 2,369 | 11,805 | 5,838 | 10,164 | 5,856 | 34,077 | 13,071 | 60,740 | 18,859 |
| *Oryzias latipes* | 4,244 | 2,117 | 11,819 | 5,690 | 10,145 | 5,781 | 33,803 | 12,104 | 60,011 | 17,388 |
| *Takifugu rubripes* | 4,223 | 2,144 | 11,979 | 5,969 | 9,826 | 6,152 | 34,540 | 16,889 | 60,568 | 23,607 |
| *Tetraodon nigroviridis* | 4,161 | 2,028 | 11,740 | 5,440 | 9,516 | 5,451 | 33,227 | 12,041 | 58,644 | 16,784 |
| Total | 12,310 |  | 24,023 |  | 27,754 |  | 62,849 |  | 84,652 |  |

**Supplementary Table S2. Primer sequences for characterizing polymorphisms.**

| Locus | Forward Primer | Reverse Primer | Contig | Annotation |
| --- | --- | --- | --- | --- |
| HtaC1002 | AGTAGGAACTCTGGTCCGCT | TTTTCTTGGTGCAGCACTGG | HAGJ01002821 | XP_014011413.1 |
| HtaC1003 | GGAGAAAGTGGTCTGCCTCT | GATGGACTGGTGTGACTGGG | HAGJ01007387 |  |
| HtaC1004 | CCCTCCTACTCCTGTCCTCC | TCATAGCAACCATCCGGCAG | HAGJ01010795 | XP_014072295.1 |
| HtaC1005 | ACTCTCTCCTCTCAGCCTCG | TGCTGTGACGTTTTAGTCCCA | HAGJ01013175 |  |
| HtaC1006 | CCCCACGTAAGAACCAGCAT | CCAACAGCAACAAGTCCCAG | HAGJ01013175 |  |
| HtaC1008 | CTGTGTGAGGCCAAGTGTCT | TCACAAGTAGAGGGGAGGGG | HAGJ01014302 |  |
| HtaC1009 | AGATGTGAAGCGACAGGACG | CTGGTCATGCTGCTGGTGTA | HAGJ01014657 | XP_013998577.1 |
| HtaC1010 | TGTGTGGGAGAATCATTGCG | GCCATGTTACTTGGTGTGCA | HAGJ01014803 |  |
| HtaC1011 | GATCATGGCCGACTCCTAGC | ACAGTGTGCTGGATCTGAGC | HAGJ01015267 | XP_014004756.1 |
| HtaC1012 | ACACTGATGCTGGACTGACC | CCATCTCAGCCACTCAGAGG | HAGJ01015382 |  |
| HtaC1013 | AGGCGCTATGTCATGTCACC | CGCCATTTCAGAACTGTGTCG | HAGJ01016741 |  |
| HtaC1015 | ATCTTGCCAGTTCCATGGCT | TCAAACTGGCCTCACATGGG | HAGJ01017397 |  |
| HtaC1016 | ATTCTTTCCTCTCCGCTCGA | AGCGAGCCTCCAACAGAATC | HAGJ01019462 |  |
| HtaC1017 | GACTACGTCGCTCTCCACTG | TGTGTGCAGTCCAACCTCTC | HAGJ01022766 |  |
| HtaC1019 | AGGTACAGATGCCTACAGGGA | TGTTGCTGCTGTTGACGTTG | HAGJ01025773 |  |
| HtaC1020 | ACCCAGCATCGCATCAGAAT | CACACATCTGCTCCCTCTGG | HAGJ01026669 |  |
| HtaC1024 | CGTCCAGTGGAGTCAGCTTT | TCGAGGACCCGCATTGAAAA | HAGJ01033063 |  |
| HtaC1025 | CACCCACACCCATGACATCA | AGAGTTAGTGGGCGTTAGCG | HAGJ01035193 | XP_014000220.1 |
| HtaC1026 | GACCGGAAGTCGACAGGAAA | CTCTCTGGTCTAAGGCTCGC | HAGJ01048208 |  |
| HtaC1027 | CCAGACCCTGGCTATGTGAA | CGCGGCTAAAGAATGACAGG | HAGJ01050464 |  |
| HtaC1028 | GCTAGTGGCATATGCGTGGA | AGCGGTTGCCCATTCATACA | HAGJ01063656 |  |
| HtaC1029 | ACTGCCATAAATACTGCGCA | AGGCAATCTGAGCTGTGACA | HAGJ01081186 |  |
| HtaC1030 | AGGACGTGGAGAACAACGTT | TGCCAATCAGCAACTCCACA | HAGJ01083920 |  |
| HtaC1031 | AGAGTCAGCACGGGAAATGG | GGCTATCTTGGCGGAGTTGT | HAGJ01090196 | XP_014051884.1 |
| HtaC1032 | TGCTGATTCTGGGTCCTGTG | TGGTTTGGCCTGAAACGTTT | HAGJ01097814 |  |
| HtaC1033 | AGACTGAACCACTGGAGACG | GCAGCAGACACGTCACTACT | HAGJ01101002 |  |
| HtaC1034 | TGTAGCTGGAACACTCAGGC | AGAATCTTCTCCCCTGGATACA | HAGJ01102768 |  |
| HtaC1035 | TGCATCACATCACTCTGCAGT | GCAAAGAATCTGCCAGCTGC | HAGJ01107218 |  |
| HtaC1036 | AAGACATGGCATCGTGGGAG | ATGCGCTTGCCTGCTTGA | HAGJ01107806 | XP_013983930.1 |
| HtaC1038 | ATCCAGAGCACACAATCGCA | TGCTACATACGAGGGGAGGG | HAGJ01111321 |  |
| HtaC1041 | ACTTGGGCATTGGTAAAAGCT | AGGCATCATGAGAGTGCGTG | HAGJ01128737 |  |
| HtaC1042 | TGCTTAGCATCAATCAAGCATGA | TCTGGCTTTCTGTTATTTCCATGT | HAGJ01129589 |  |
| HtaC1043 | GCTTGCGAGAACTCTGTCCT | CTGCCAATGAAGAGCGTGTG | HAGJ01129704 |  |
| HtaC1044 | ACGTAGCTAGACGACCACCA | GGCATGCGTTTCGGTGTATT | HAGJ01129741 | XP_013986860.1 |
| HtaC1045 | CCAACAGCCTCCGATAGACC | CTGTCTTTTCCCTCCCGGTC | HAGJ01130713 |  |
| HtaC1046 | TTGAAGATTGCCGTTGCTGC | TGAGTAGCAAGTTGGCCCTG | HAGJ01133634 |  |
| HtaC1047 | GTTCTCACTCTCTGCCGCAT | GTGTAGCCTGCTAAGACGCA | HAGJ01138392 |  |
| HtaC1048 | CTTCTCTTCTCTCGCGCACA | GCCCTTAGACCAGGCTTTGA | HAGJ01140428 | NP_001133406.1 |
| HtaC1049 | TCCCTTCCAGCTATTTGCCG | CATGGGCCTGTCTTTGCAAC | HAGJ01143928 |  |
| HtaC1050 | AGTAACCTGGAACCTTGCCG | TGGAATCCGGAATCCTGTGC | HAGJ01144708 |  |
| HtaC1051 | GTGACAAAGGCCTCGAGTGA | GCCTATCACCATGGCAACCT | HAGJ01144732 | XP_014037089.1 |
| HtaC1053 | TCCGGTTGTTCCCATTGACC | TCGGTTAAATTGTGCAAAGCTGA | HAGJ01164514 | XP_013997324.1 |

**Supplementary Table S3.** Sequence quality assessment of taimen transcripts.

| Gene | Taimen transcript | Identity | Alignment length | E-value | Query length | Target length |
| --- | --- | --- | --- | --- | --- | --- |
| ND1 | HAGJ01008946 | 99.69 | 972 | 0 | 972 | 13135 |
| ND2 | HAGJ01008946 | 99.62 | 1048 | 0 | 1048 | 13135 |
| COX1 | HAGJ01008946 | 100 | 1551 | 0 | 1551 | 13135 |
| COX2 | HAGJ01008946 | 99.71 | 691 | 0 | 691 | 13135 |
| ATP8 | HAGJ01008946 | 100 | 168 | 3.00E-84 | 168 | 13135 |
| ATP6 | HAGJ01008946 | 100 | 684 | 0 | 684 | 13135 |
| COX3 | HAGJ01008946 | 99.87 | 785 | 0 | 785 | 13135 |
| ND3 | HAGJ01015371 | 93.43 | 350 | 4.00E-146 | 349 | 2623 |
| ND4L | HAGJ01015371 | 100 | 297 | 1.00E-155 | 297 | 2623 |
| ND4L | HAGJ01015371 | 99.78 | 1357 | 0 | 1381 | 2623 |
| ND5 | HAGJ01157312 | 99.46 | 1839 | 0 | 1839 | 2699 |
| ND6 | HAGJ01157312 | 91 | 522 | 0 | 522 | 2699 |
| CYTB | HAGJ01156979 | 99.91 | 1098 | 0 | 1141 | 1100 |
| KU049783 | HAGJ01021905 | 100 | 453 | 0 | 453 | 1366 |
| KU049782 | HAGJ01131345 | 100 | 367 | 0 | 367 | 1879 |
| KU049781 | HAGJ01152928 | 99.5 | 599 | 0 | 599 | 2867 |
| KU049780 | HAGJ01004592 | 94.14 | 256 | 7.00E-108 | 256 | 680 |
| KU049779 | HAGJ01145988 | 99.61 | 255 | 1.00E-130 | 255 | 2152 |
| KU049778 | HAGJ01015081 | 98.35 | 423 | 0 | 425 | 1007 |
| KU049777 | HAGJ01116327 | 100 | 286 | 2.00E-149 | 286 | 835 |
| KU049776 | HAGJ01122448 | 100 | 412 | 0 | 412 | 1707 |
| KU049775 | HAGJ01028091 | 99.56 | 681 | 0 | 681 | 2500 |
| KU049774 | HAGJ01143160 | 99.54 | 431 | 0 | 488 | 1163 |
| KU049773 | HAGJ01070686 | 99.75 | 395 | 0 | 395 | 768 |
| KU049772 | HAGJ01125350 | 97.38 | 229 | 9.00E-107 | 263 | 1056 |
| KU049771 | HAGJ01168534 | 99.87 | 792 | 0 | 794 | 1102 |
| KU049770 | HAGJ01078835 | 99.75 | 396 | 0 | 396 | 489 |
| KU049769 | HAGJ01161051 | 97.1 | 723 | 0 | 731 | 2353 |
| KR106990 | HAGJ01122494 | 99.65 | 572 | 0 | 573 | 1039 |
| KP137362 | HAGJ01176354 | 100 | 645 | 0 | 645 | 910 |
| KC508833 | HAGJ01000607 | 100 | 770 | 0 | 792 | 770 |
| KF554113 | HAGJ01115027 | 99.7 | 2017 | 0 | 2737 | 2016 |
| JF951962 | HAGJ01017368 | 82.14 | 112 | 1.00E-17 | 1444 | 1659 |

**Supplementary Table S4. Assessment of transcriptome shotgun assembly using BUSCO.** Single-copy orthologs from the Actinopterygii lineage were adopted for the assessment.

| Category | Single copy orthology | Percentage | Taimen transcripts |
| --- | --- | --- | --- |
| Single-copy complete orthologs | 1371 | 29.9% | 1371 |
| duplicated complete orthologs | 1189 | 25.9% | 2847 |
| Fragmented orthologs | 867 | 18.9% | 867 |
| Missing orthologs | 1156 | 25.2% |  |
| Total | 4584 |  |  |

**Supplementary Table S5. Summary of the KEGG pathway annotations of the taimen transcriptome**

(Included in a separated excel file)

**Supplementary Table S6. Positive selection transcripts in taimen**

(Included in a separated excel file)

**Supplementary Table S7. Positive selection test with the Branch_Site model**

(Included in a separated excel file)

**Supplementary Figures**


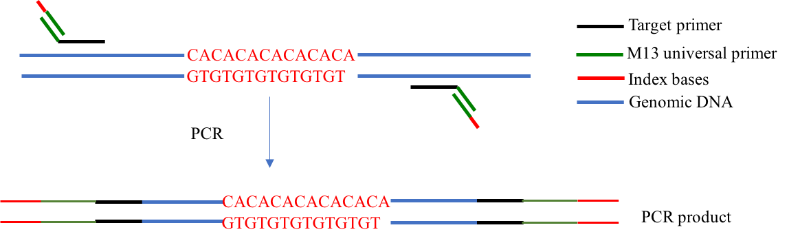


**Supplementary Figure S1. Illustration of the microsatellite primers used to characterize polymorphisms.** Target primers were used to amplify target genomic DNA, and index primers (contained index bases and M13 universal primer sequences) were used to add index bases for the samples. The M13 universal primer sequences are listed in the Supplementary Text.


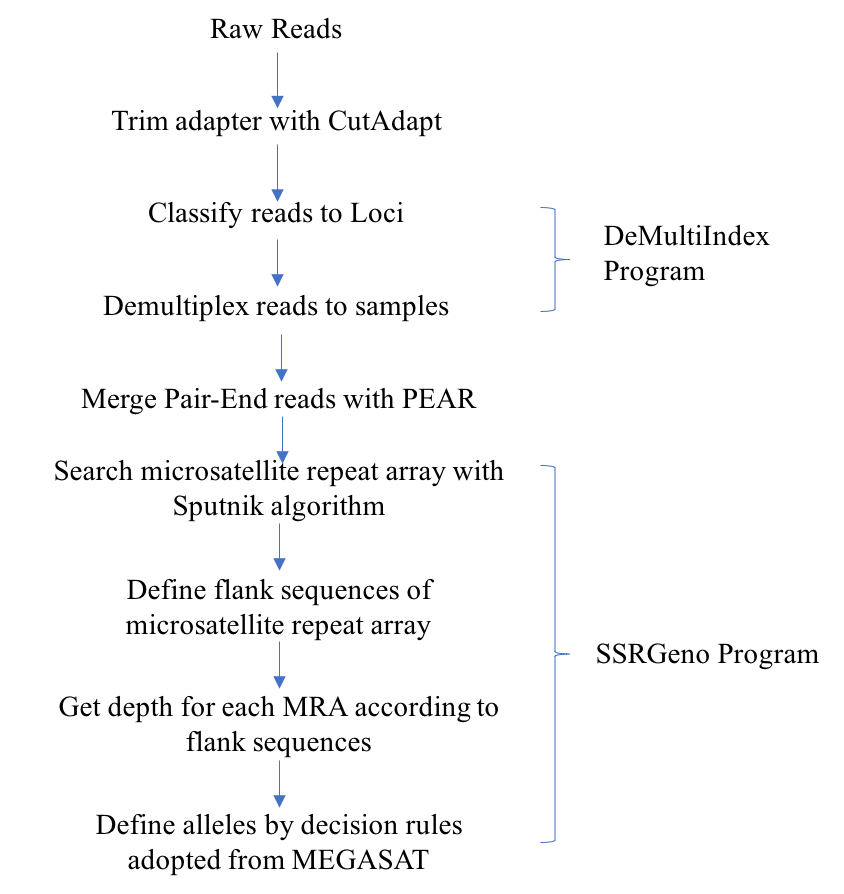


**Supplementary Figure S2. Flow chart of the pipeline used to define microsatellite genotypes.**


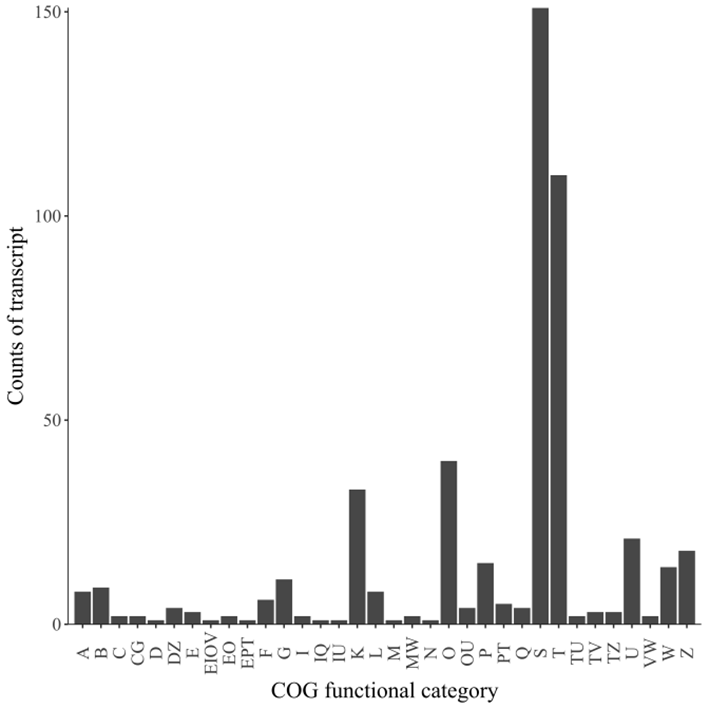


**Supplementary Figure S3. COG functional category of transcripts within 313 gene families occurring only in taimen.**


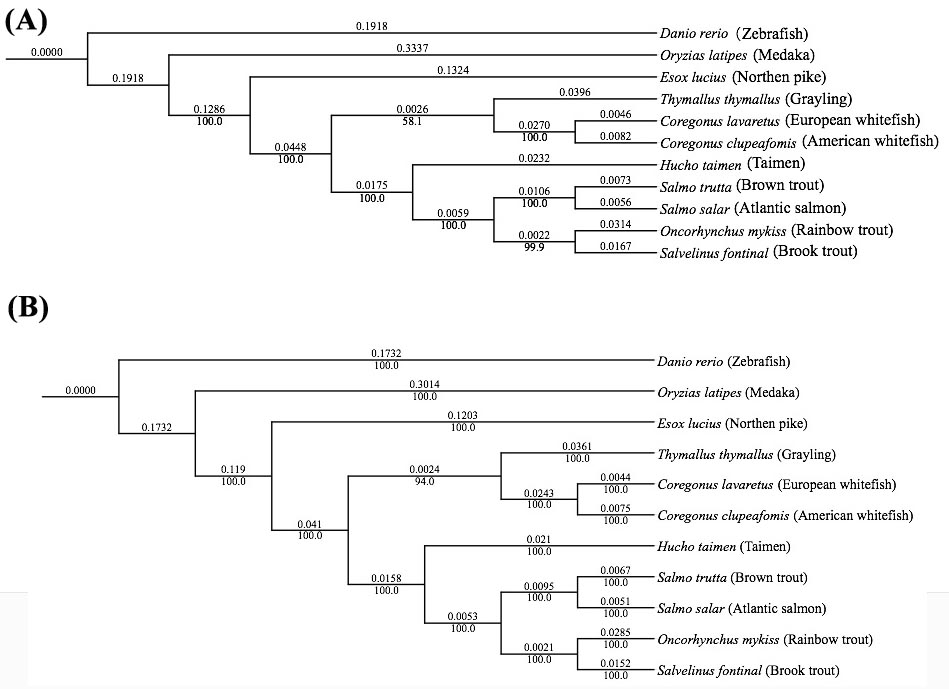


Supplementary Figure S4. Phylogenetic trees inferred by PhyML(A) and MrBayes(B). The topological structures were consistent. Numbers under branches are bootstrap values (%), numbers above branches are branches length inferred by programs.


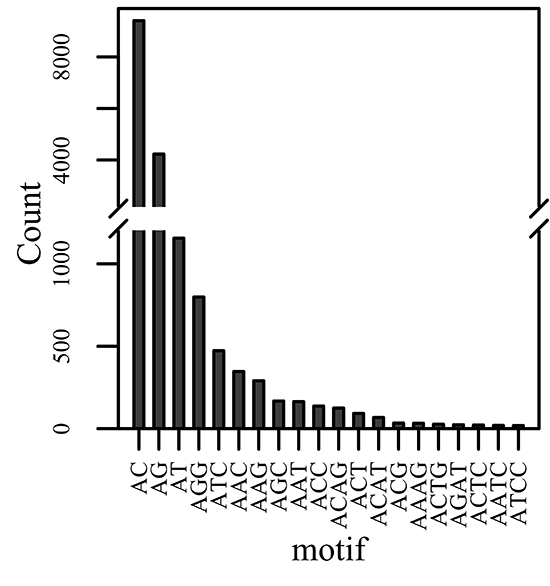


**Supplementary Figure S5. Abundance of the top 20 motifs**


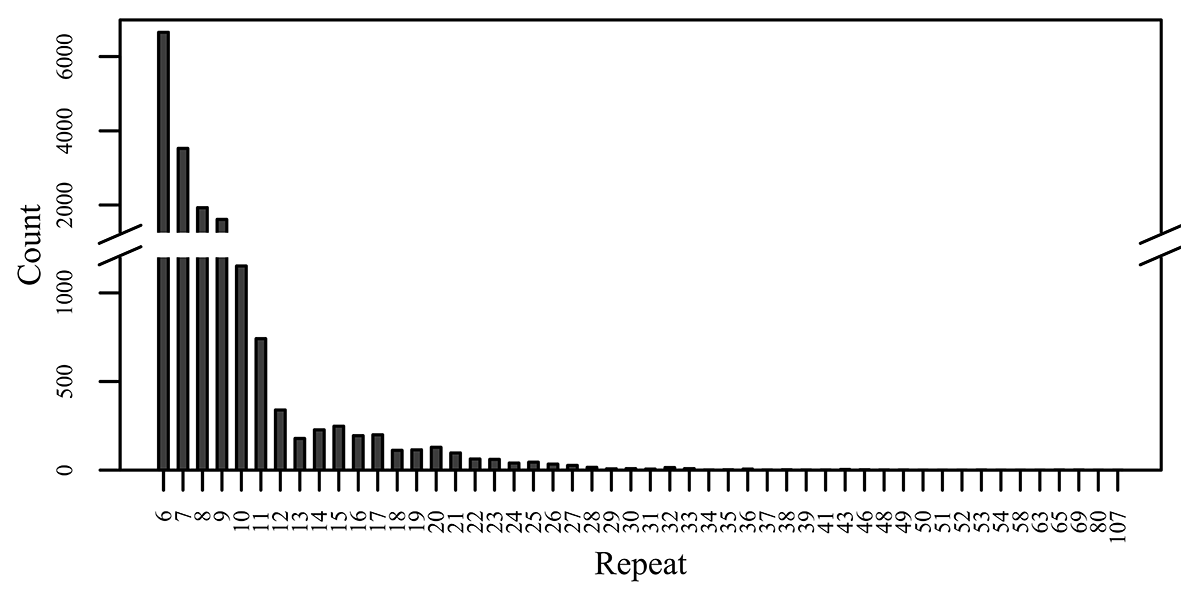


**Supplementary Figure S6. Abundance of the top 20 repeats**


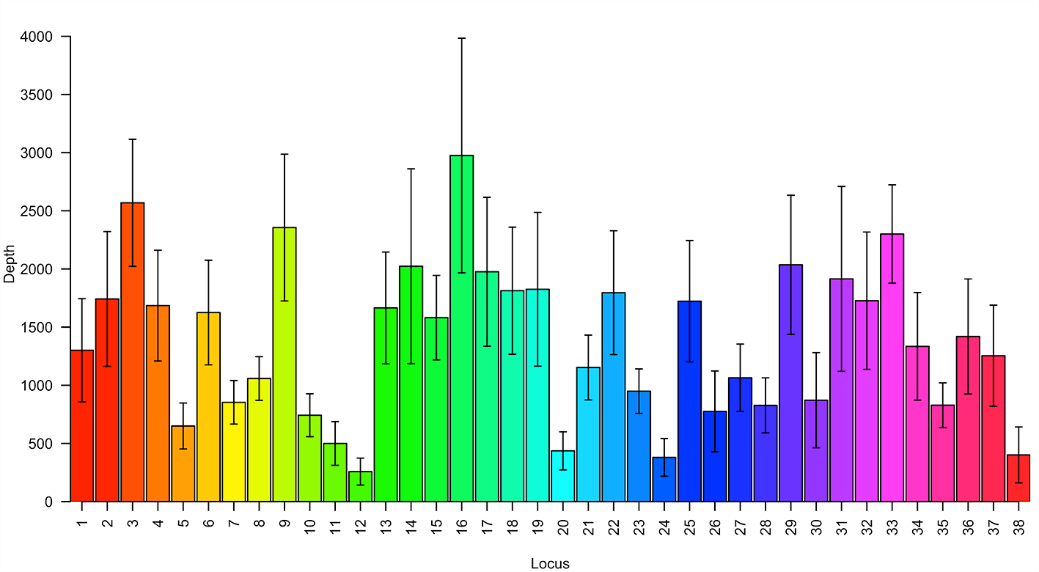


**Supplementary Figure S7. Sequencing depth of 38 loci.** The X axis shows sequencing loci, and the Y axis shows the mean count of sequencing reads of all 32 samples in each marker; error bars represent the standard variation of the reads count.


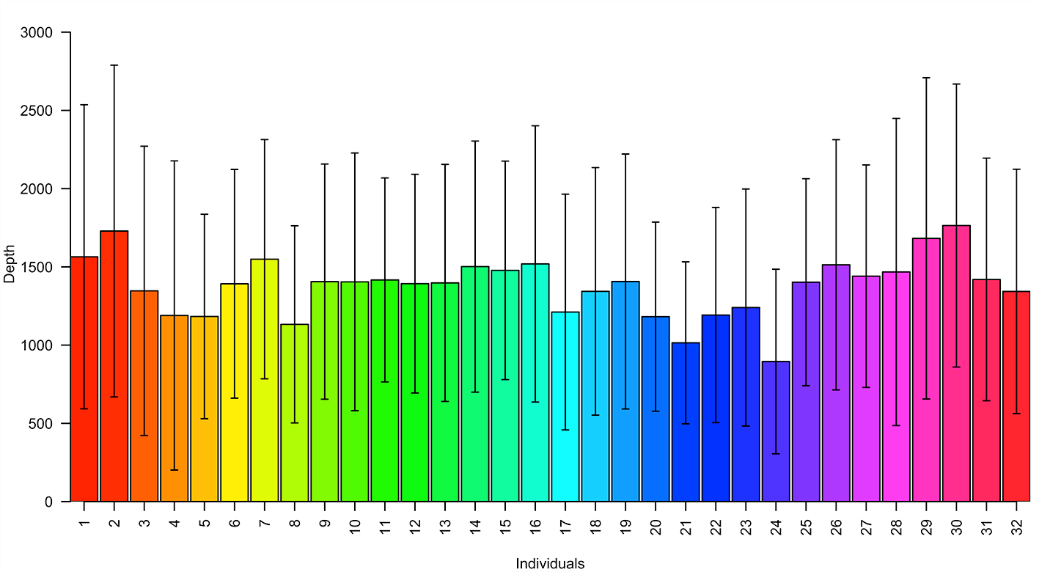


**Supplementary Figure S8. Sequencing depth of 32 samples.** The X axis shows 32 samples, and the Y axis shows the mean count of sequencing reads for all 38 markers in each sample; error bars represent the standard variation of the reads count.

**References**

La Rota, M., Kantety, R. V., Yu, J. K., & Sorrells, M. E. (2005). Nonrandom distribution and frequencies of genomic and EST-derived microsatellite markers in rice, wheat, and barley. *BMC Genomics*, *6*, 23. http://doi.org/10.1186/1471-2164-6-23

Martin, M. (2011). Cutadapt removes adapter sequences from high-throughput sequencing reads. *EMBnet.Journal*, *17*(1), pp. 10–12. http://doi.org/10.14806/ej.17.1.200

Zhan, L., Paterson, I. G., Fraser, B. A., Watson, B., Bradbury, I. R., Nadukkalam Ravindran, P., et al. (2016). MEGASAT: automated inference of microsatellite genotypes from sequence data. *Molecular Ecology Resources*, *17*(2), 247–256. http://doi.org/10.1111/1755-0998.12561

Zhang, J., Kobert, K., Flouri, T., & Stamatakis, A. (2014). PEAR: a fast and accurate Illumina Paired-End reAd mergeR. *Bioinformatics*, *30*(5), 614–620. http://doi.org/10.1093/bioinformatics/btt593
